# 

**Published:** 1877-06-20

**Authors:** 


☞ All communications relating to the business of The Record, for the year 1877, must be addressed to

DR. R. C. WORD,

Business Manager Southern Med. Rec.,

Atlanta, Ga.

☞ Brief and practical communications are solicited on all subjects pertaining to medicine, also reports of cases in practice.

☞ Send money by check, postal order, or registered letter.

☞ Write your name, post-office, county and State plainly.

☞ Our senior editor, Dr. T. S. Powell, was appointed a delegate to the late meeting of the American Medical Association, but was unable to attend by reason of sickness in his family.

### COLLABORATORS AND CONTRIBUTORS.

L. B. Anderson, M.D., Va.
Swan M. Burnett, M.D., D. C
J. M. Bristow M.D., Texas
J. W. Booth, M.D., N. C.
W. F. Barr, M.D., Va.
B. W. Corn, M.D., Texas.
John Castor, M.D., Iowa.
E. T. Easley. A.M., M.D., Ark.
A. H. Goelet, M D., N. Y.
E. C. Gehrung, M.D., Mo.
G. D. Hodge, M.D., Ark.
S. M. Hogan, M.D., Ala.
A. McLane Hamilton, M.D.,N.Y.
C. C. Vanderbeck, M.D., Pa.
Z. B. Herndon, M.D., Va.
Lindsav Johnson, M.D., Ga.
A. R. Kilpatrick, M.D., Tex.
R. Kells, M.D., Miss.
W. L. Lipscomb, M.D., Miss.
J. M. Lewis, M.D., Miss.
G. A. Dyer, M.D., Ind.
C. H. Lathrop, M.D., Iowa.
A. A. Lyon, M.D.. La.
M. Lewis, M.D., Tenn.
G. M. Rivers, M.D., S. C.
A. S. Payne, M.D., Va.
A. M. Whitehead, M.D., O.

